# Collagen Xerogel Thread Associated With Reduced Inflammatory and Fibrotic Histological Changes During Early Urethral Wound Healing in Rats

**DOI:** 10.7759/cureus.110658

**Published:** 2026-06-11

**Authors:** Shohei Tobu, Maki Kawasaki, Toshiaki Takezawa, Kei Nagase, Megumi Nishiyama, Akihiro Maeda, Shigehisa Aoki, Mitsuru Noguchi

**Affiliations:** 1 Department of Urology, Saga University Faculty of Medicine, Saga, JPN; 2 Department of Pharmacology, Chiba Institute of Science, Chiba, JPN; 3 Department of Pathology, Saga University Faculty of Medicine, Saga, JPN

**Keywords:** collagen xerogel thread, fibrosis, inflammation, urethra, wound healing

## Abstract

Background

Early inflammatory and fibrotic responses play important roles in tissue remodeling during wound healing. We developed a novel high-density collagen xerogel thread (CXT) and evaluated its effects on early urethral tissue responses in a rat urethral injury model.

Methods

A rat urethral incision model was used to compare CXT with conventional absorbable Vicryl® sutures (Ethicon, Inc., Somerville, NJ, USA). Histological evaluation was performed using hematoxylin-eosin staining and immunohistochemical analyses for alpha-smooth muscle actin (α-SMA), connective tissue growth factor (CTGF), and leukocyte common antigen (LCA) on postoperative day 7.

Results

Compared with Vicryl sutures, CXT was associated with reduced urethral mucosal thickening, lower numbers of CTGF-positive cells and LCA-positive cells, and less prominent stromal α-SMA expression during early wound healing.

Conclusions

CXT was associated with altered inflammatory and fibrotic tissue responses during early urethral wound healing in rats.

## Introduction

Urethral wound healing is a complex biological process involving inflammation, tissue remodeling, and extracellular matrix deposition [1,2]. Excessive inflammatory and fibrotic responses during wound healing may adversely affect tissue architecture and repair quality [3,4]. Therefore, understanding factors that influence early tissue responses is important for improving surgical wound healing outcomes.

Suture materials are indispensable components of reconstructive surgery and may influence local tissue reactions, including inflammation, foreign-body response, and fibrosis [5,6]. Previous studies have suggested that material composition and structural characteristics of sutures can affect wound healing and tissue remodeling [5,6]. However, the impact of different suture materials on early urethral tissue responses remains incompletely understood.

Collagen-based biomaterials have attracted considerable interest because of their potential to support tissue regeneration and modulate inflammatory responses [7,8]. We previously developed a high-density collagen material, Vitrigel, which has demonstrated favorable biological properties in several regenerative applications, including skin repair and fibrosis-related models [9-14]. Collagen xerogel thread (CXT), a thread-type formulation of this material, was developed as a novel collagen-based suture material that may provide structural support while minimizing excessive tissue reactions [12].

The aim of this study was to evaluate the effects of CXT on early inflammatory and fibrotic tissue responses during urethral wound healing compared with conventional absorbable sutures using a rat urethral incision model.

## Materials and methods

Collagen xerogel thread (CXT)

**Figure 1:**
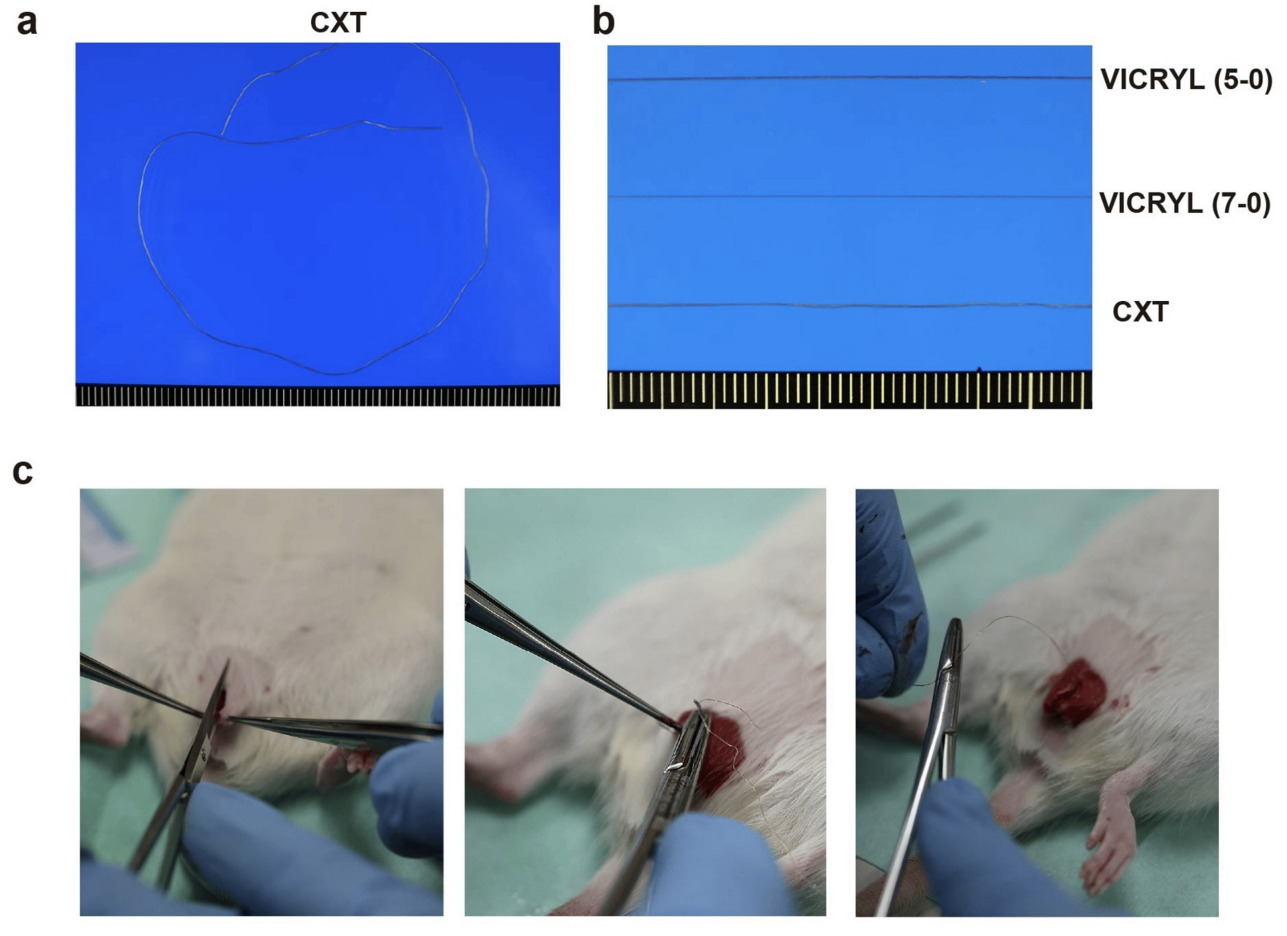
Surgical application of collagen xerogel thread (CXT) (a) CXT is a high-density collagen-based biomaterial. (b) Comparison of thread diameter between CXT and Vicryl 5-0 sutures. The diameter of CXT was comparable to that of synthetic absorbable sutures. (c) Urethral surgical procedure using CXT. A 7-mm longitudinal incision was created at the 12 o’clock position of the urethra. The incision was closed with four interrupted sutures using CXT and a 3/8 circle taper needle.

CXTs were prepared as previously described [12]. Briefly, a 0.5% atelocollagen sol was prepared by mixing equal volumes of a 1.0% acidic solution of porcine-derived atelocollagen (Nippon Meat Packers Inc., Tokyo, Japan) and serum-free culture medium. The sol was gelled to form columnar collagen gels, which were subsequently vitrified by ultraviolet irradiation and dehydration. Following rehydration and revitrification, CXTs were obtained and further sterilized by ultraviolet irradiation (Figure 1A). The diameter of the CXT was comparable to that of commercially available absorbable Vicryl® 5-0 sutures (polyglactin 910; Ethicon Inc., Somerville, NJ, USA) (Figure 1B).

Animal model

Six-week-old female Sprague-Dawley rats (Nippon SLC Co., Ltd., Shizuoka, Japan) were used. Female rats were selected because their urethral anatomy allows more reproducible histological assessment than that of male rats. All procedures were performed under general anesthesia with isoflurane. A 7-mm longitudinal urethral incision was created at the 12 o’clock position from the external urethral meatus while gently applying traction to the urethra (Figure 1C). The incision was closed with four interrupted sutures using the designated suture material and a 3/8-circle taper needle.

The animals were assigned to five experimental groups: (1) sham (no incision), (2) nonsutured incision, (3) Vicryl® 5-0 suture, (4) Vicryl® 7-0 suture, and (5) CXT suture. Vicryl sutures were selected as representative absorbable sutures commonly used in clinical practice. Suturing was performed only on the urethra, and the overlying skin was left unsutured.

Periurethral tissues were harvested en bloc on postoperative day 7. Histological evaluation was performed using hematoxylin-eosin staining and immunohistochemistry for alpha-smooth muscle actin (α-SMA), connective tissue growth factor (CTGF), and leukocyte common antigen (LCA). Urethral mucosal thickness and the numbers of CTGF-positive and LCA-positive cells were quantified in predefined periurethral regions of interest. α-SMA expression was evaluated qualitatively. Representative images were selected from anatomically comparable periurethral regions. Five animals were included in each group.

Statistical analysis was performed using one-way ANOVA followed by Tukey's multiple comparison test. Data are presented as mean ± standard deviation (SD). A p-value < 0.05 was considered statistically significant. Sample size was determined based on our previous exploratory animal studies and practical considerations.

All animal experiments were approved by the Animal Experimentation Committee of Saga University (Approval No. A2021-011-1).

## Results

Macroscopic changes in the urethra

**Figure 2:**
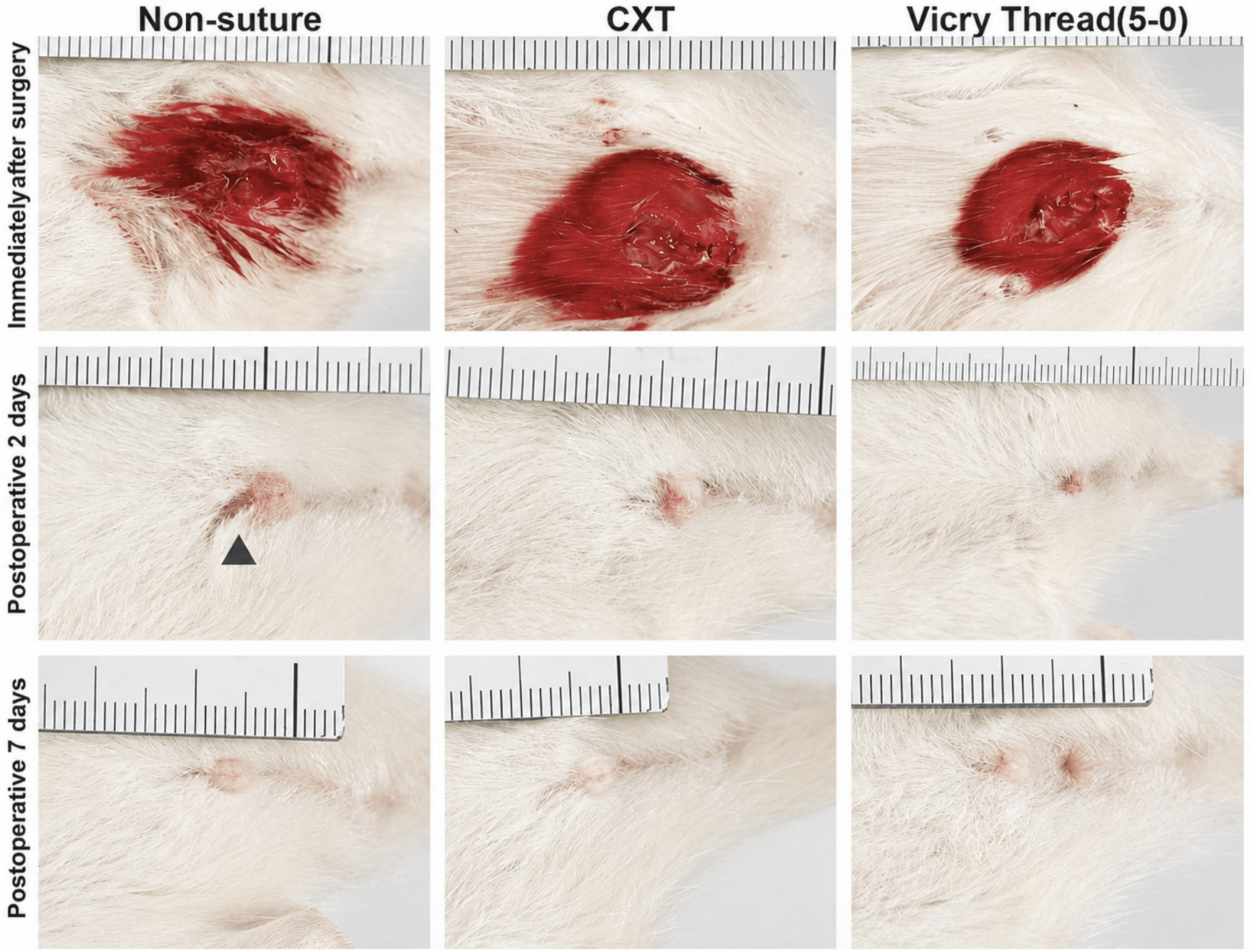
Macroscopic appearance of the urethra after surgery On postoperative day 2, the nonsutured group showed delayed wound healing compared with the CXT and Vicryl groups (black arrowhead). By day 7, the CXT group showed smooth tissue recovery, whereas the Vicryl group exhibited apparent tissue irregularity at the surgical site. CXT: collagen xerogel thread

Wound healing was observed from postoperative day 2 in all groups. The nonsutured group showed delayed healing compared with the CXT and Vicryl groups. By postoperative day 7, the CXT group showed smooth tissue recovery, whereas the Vicryl group exhibited tissue irregularity at the surgical site (Figure 2). Histological evaluation was therefore performed on postoperative day 7.

Urethral mucosal thickness

**Figure 3:**
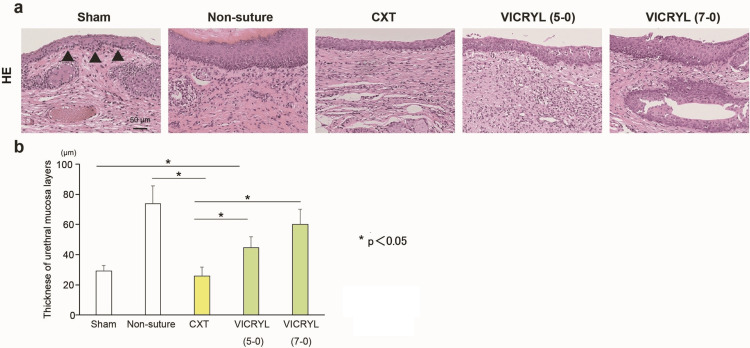
Urethral mucosal thickness (a) Representative hematoxylin-eosin-stained sections of the urethral mucosa in the sham, nonsutured, collagen xerogel thread (CXT), Vicryl 5-0, and Vicryl 7-0 groups on postoperative day 7. (b) Quantitative analysis of urethral mucosal thickness. Data are presented as mean ± SD (n = 5 animals per group). Statistical comparisons were performed using one-way ANOVA followed by Tukey's multiple comparison test. *P < 0.05.

The nonsutured group showed the greatest degree of urethral mucosal thickening. Urethral mucosal thickness was significantly lower in the CXT group than in the Vicryl group (Figure 3).

Myofibroblast-related changes (α-SMA)

**Figure 4:**
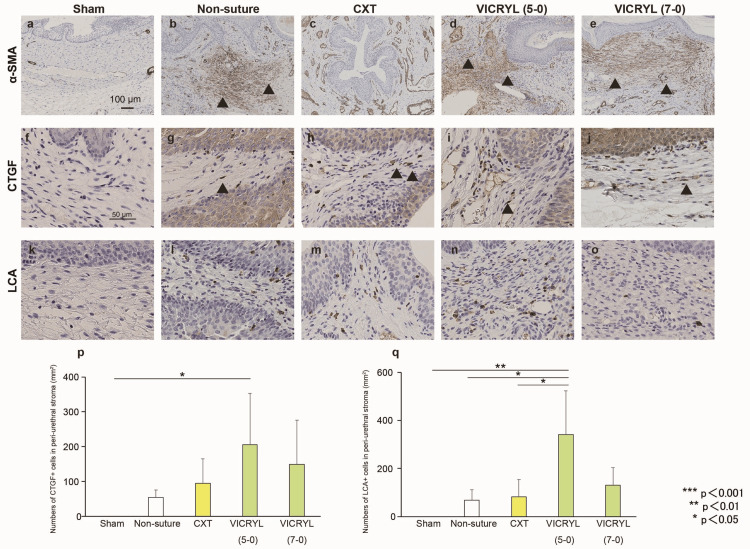
Immunohistochemical analysis of fibrosis- and inflammation-related markers in periurethral tissue (a-e) α-SMA immunostaining. Diffuse stromal α-SMA staining was observed in the nonsutured and Vicryl groups, whereas minimal staining was observed in the sham and CXT groups. (f-j) CTGF immunostaining. CTGF-positive cells were more frequently observed in the Vicryl groups than in the sham and CXT groups. (k-o) LCA immunostaining. LCA-positive cells were more frequently observed in the Vicryl groups than in the sham and CXT groups. (p) Quantitative analysis of CTGF-positive cells. (q) Quantitative analysis of LCA-positive cells. Representative images were selected from anatomically comparable periurethral regions. Quantification was performed in predefined periurethral regions of interest. Black arrowheads indicate positive cells. Data are presented as mean ± SD (n = 5 animals per group). *P < 0.05, **P < 0.01. CXT: collagen xerogel thread; α-SMA: alpha-smooth muscle actin; CTGF: connective tissue growth factor; LCA: leukocyte common antigen

Diffuse stromal α-SMA staining was observed in the nonsutured and Vicryl 5-0 groups, whereas minimal staining was observed in the sham and CXT groups (Figures 4a-4e).

Fibrotic marker expression (CTGF)

CTGF-positive cells were more frequently observed in the Vicryl 5-0 group than in the sham and CXT groups. The number of CTGF-positive cells appeared lower in the CXT group than in the Vicryl 5-0 group (Figures 4f-4j, 4p).

Inflammatory cell infiltration (LCA)

LCA-positive cells were increased in the Vicryl group compared with the other groups. The number of LCA-positive cells appeared lower in the CXT group than in the Vicryl group (Figures 4k-4o, 4q).

## Discussion

The present study evaluated early tissue responses to a novel collagen-based suture material in a rat urethral injury model. Histological and immunohistochemical analyses demonstrated reduced urethral mucosal thickening and lower expression of fibrosis- and inflammation-related markers in the CXT group compared with the Vicryl group.

Inflammation and myofibroblast activation are key processes during wound healing and are closely associated with tissue remodeling and fibrosis [15,16]. CTGF is a well-established profibrotic mediator involved in extracellular matrix deposition and scar formation during tissue repair [17]. LCA (CD45) is widely used as a marker of inflammatory leukocyte infiltration and has been employed to assess local inflammatory responses in various experimental models [18].

In the present study, diffuse stromal α-SMA expression, increased CTGF-positive cells, and increased LCA-positive cell infiltration were observed more frequently in the Vicryl group than in the CXT group.

Previous studies have demonstrated that collagen-based biomaterials can modulate tissue repair and reduce excessive fibrosis in various experimental settings [11-14]. Previous studies using CXT have also demonstrated reduced inflammatory and fibrotic tissue reactions, suggesting favorable biocompatibility and low immunogenic potential [12]. Consistent with these observations, the present findings suggest that the biological properties of CXT may contribute to differences in local tissue responses compared with conventional absorbable sutures [11-14].

Limitations

This study should be considered a preliminary pilot study and has several limitations. First, the sample size was relatively small. Second, evaluation was limited to postoperative day 7, and long-term outcomes were not assessed. Therefore, the present findings should not be interpreted as evidence of reduced fibrosis, improved wound healing, or prevention of urethral stricture. Third, this model represents an acute wound-healing model rather than a validated urethral stricture model. Fourth, a clinically established monofilament comparator such as polydioxanone (PDS) was not included, and differences in filament structure may have influenced the observed tissue responses. Finally, the molecular mechanisms underlying the observed histological differences were not investigated.

Despite these limitations, the present study provides preliminary histological evidence that CXT may alter early inflammatory and fibrotic tissue responses following urethral injury. Further studies incorporating long-term evaluation, functional assessment, and clinically relevant experimental models are warranted to clarify the biological and translational significance of these findings.

## Conclusions

CXT was associated with reduced inflammatory cell infiltration and decreased expression of fibrosis-related markers during the early phase of urethral wound healing in rats. However, the present findings are based on a small, short-term animal study and should be interpreted with caution. Further studies using long-term and clinically relevant models are warranted to clarify its potential role in urethral reconstruction.
